# Optimized Graphene-Oxide-Based Interconnecting Layer in All-Perovskite Tandem Solar Cells

**DOI:** 10.1021/acsenergylett.4c03065

**Published:** 2025-01-09

**Authors:** Melissa R. Fitzsimmons, Bart Roose, Yutong Han, Taeheon Kang, Yu-Hsien Chiang, Chieh-Szu Huang, Yang Lu, Terry Chien-Jen Yang, Cullen Chosy, Shaoliang Guan, Miguel Anaya, Samuel D. Stranks

**Affiliations:** † Department of Chemical Engineering and Biotechnology, University of Cambridge, Cambridge CB3 0AS, United Kingdom; ‡ Department of Physics, Cavendish Laboratory, University of Cambridge, Cambridge CB3 0HE, United Kingdom

## Abstract

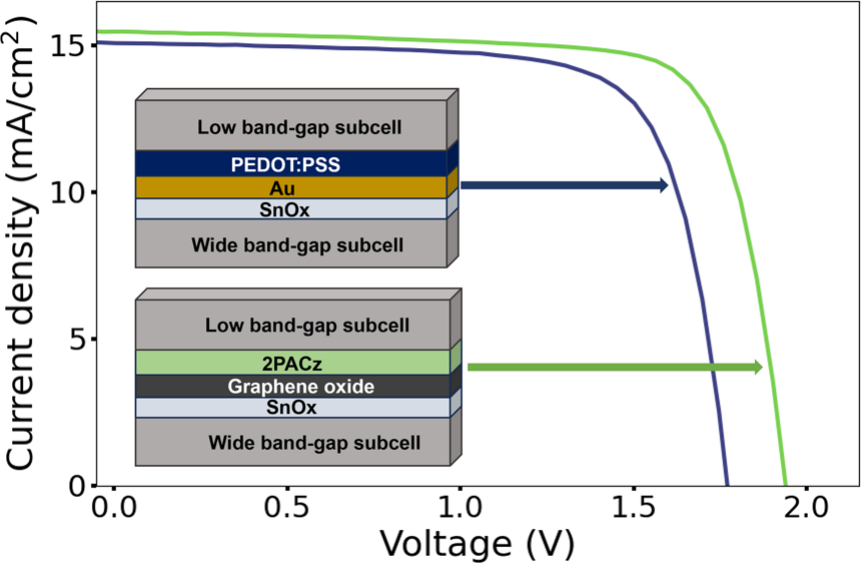

All-perovskite tandem solar cells represent a significant advancement in next-generation photovoltaics toward higher power conversion efficiencies than single junction cells. A critical component of a monolithic tandem solar cell is the interconnecting layer, which facilitates the integration of the wide bandgap and low bandgap subcells. Conventional designs in all-perovskite tandem cells are based on an ultrathin metal recombination layer, typically Au, alongside a poly(3,4-ethylenedioxythiophene):polystyrenesulfonate (PEDOT:PSS) hole transporting layer, which introduce optical and recombination losses, and instabilities. Here, we present a new interconnecting layer based on a graphene-oxide recombination layer, which facilitates the replacement of PEDOT:PSS with the preferred self-assembled monolayer [2-(9*H*-carbazol-9-yl)ethyl]phosphonic acid (2PACz). This device architecture results in significantly reduced optical and nonradiative losses, leading to champion device efficiency of 23.4% compared to 19.7% with the conventional layers, along with improvements in stability. This work solves a critical challenge in all-perovskite tandem cell device design.

Tandem solar cells (TSCs) represent a promising avenue for enhancing the photovoltaic efficiency beyond the Shockley–Queisser limit of single-junction solar cells. Halide perovskites have excellent optoelectronic properties such as high absorption coefficients, long carrier diffusion lengths and charge-carrier lifetimes.^1−3^ All-perovskite TSCs consist of a wide-bandgap (WBG) perovskite top cell and a low-bandgap (LBG) perovskite bottom cell that absorb complementary regions of the solar spectrum. All-perovskite cells have been demonstrated to achieve a certified record power conversion efficiency (PCE) of 30.1%, surpassing that of certified single-junction perovskite solar cells (26.7%).^4^ A range of mixed lead–tin perovskite compositions has enabled low band gaps of 1.22–1.25 eV to be achieved,^5−7^ which typically pair with an optimum top cell band gap of 1.75–1.85 eV in a tandem device.^8^ Device and optical simulations predict that the theoretical PCE limit of >40% can be reached using currently available materials with bandgaps of 1.25 eV for LBG and 1.8–1.9 eV for WBG absorber layers. When light coupling between the layers is taken into account, the optimum efficiency is maintained even when more stable WBG perovskite materials with bandgaps of 1.6–1.7 eV are used.^9^

In a two-terminal tandem device, subcells are mechanically, electrically, and optically connected by an interconnecting layer. In solution-processed perovskite tandem solar cells, the interconnecting layer must maintain a good solvent barrier to prevent damage to the underlying subcell. The most commonly investigated interconnecting layers are based on a recombination layer, which provides a recombination site for carriers extracted from each subcell, enabling rapid charge transfer and for charge neutrality to be maintained across the device.^10,11^ In the early development of perovskite TSCs, the interconnecting layer was based on a transparent conductive oxide recombination layer, which consisted of a thin layer of SnO_2_ deposited by atomic layer deposition (ALD) and a thick transparent conducting oxide layer such as indium tin oxide (ITO) of around 100–120 nm to act as a solvent barrier during processing of the top subcell.^12−15^ The high conductivity of the transparent conductive oxide can result in a high fill factor (FF) due to low resistive losses; however, the thick layer increases both the parasitic absorption and risk of shunting due to the high lateral conductivity.^10,11^

More recently, the deposition of a thicker (10–20 nm) ALD layer, most commonly SnO_2_, has been reported to act as both a buffer layer and a solvent barrier, enabling the recombination layer thickness to be reduced significantly. In state-of-the-art perovskite TSCs, the ALD-SnO_2_ layer is typically paired with either a transparent conducive oxide-based recombination layer^16,17^ or an ultrathin metal recombination layer, such as Au,^18−23^ alongside a poly(3,4-ethylenedioxythiophene):polystyrenesulfonate (PEDOT:PSS) hole transporting layer (HTL).^21,24^ Since the high-power sputtering generally required to deposit transparent conductive oxides can potentially damage the layers underneath, an ultrathin metal, deposited by thermal evaporation, has become the most common recombination layer. However, there are still significant optical losses from parasitic absorption from both the Au and the PEDOT:PSS layers. These losses limit the maximum achievable short circuit current density (*J*_SC_) in the LBG subcell, decreasing the value at which current matching can be achieved between the subcells.

Alongside optical losses, degradation in perovskite TSCs can arise from the metal-containing interconnecting layer and PEDOT:PSS hole transporting layer (HTL). The presence of metal has been found to reduce the thermal stability of perovskite solar cells as the metal species diffuse into the perovskite layer,^25−27^ while PEDOT:PSS is widely reported to facilitate the degradation of solar cells due to its acidic and hydroscopic characteristics.^28−32^ Although PEDOT:PSS remains the primary HTL in the majority of reported high-efficiency lead–tin solar cells, it has been shown to lead to severely worsened charge extraction after thermal aging in these devices.^14,33,34^ Other alternative HTLs to the PEDOT:PSS in lead–tin perovskite solar cells include PTAA^35^ and NiO_*x*_,^14,27^ which have achieved good results but suffer from poor reproducibility. Solution-processed ITO nanocrystals have been recently reported to act as both the recombination layer and the HTL in perovskite TSCs,^27,36^ which improves stability while reducing the parasitic light absorption from the traditional Au/PEDOT:PSS interlayers, though more sustainable indium-free alternatives would be desired. These reports highlight the potential in using alternative HTLs and interconnecting layers for optimizing cell performance, but working selections remain limited to date.

An alternative HTL to PEDOT:PSS is a self-assembled monolayer (SAM). Al-Ashouri et al. developed a family of carbazole-based SAMs such as [2-(9*H*-carbazol-9-yl)ethyl]phosphonic acid (2PACz).^37^ The phosphonic acid group of the SAM can bind to an oxide surface, such as ITO, forming a thin (<1 nm) monolayer. There have been a large number of reports of the use of carbazole-based SAMs in high-efficiency single-junction Pb-based perovskites, as well as in the WBG subcell of tandem devices, with studies reporting reduced nonradiative recombination losses compared to other analogues.^37−40^ Recent reports indicate that substituting PEDOT:PSS with a SAM in single junction lead–tin perovskite solar cells has led to improved PCE and enhanced stability under different conditions.^34,41^ However, despite these reports that pose the SAMs as the preferred HTL in single junction devices, SAMs are not reliably implemented as the HTL in the lead–tin LBG subcell in TSCs, and it is not yet clear why. Datta et al.^42^ reported a 2PACz HTL alongside a sputtered ITO recombination layer but a reduced PCE compared to a PEDOT:PSS HTL was obtained.

Here, we aim to understand and solve these limitations in order to leverage the demonstrated advantages of phosphonic-based SAMs within our tandem architecture. We first show that 2PACz is not suitable as the HTL in the LBG subcell when combined with the commonly used SnO_2_/Au interconnecting layer because the 2PACz binds poorly to Au and thus forms a discontinuous, ineffective HTL. Instead, we implement a new interconnecting layer in which the ultrathin Au recombination layer is replaced with graphene oxide (GO), an easily processable and sustainable layer. The abundance of oxygen-containing functional groups in the GO enables the 2PACz to bind, allowing successful replacement of the PEDOT:PSS HTL in the LBG subcell with 2PACz. We reproducibly observe a higher device *J*_SC_ and open-circuit voltage (*V*_OC_) when the new interconnecting layer is implemented. We obtain a champion power conversion efficiency (PCE) of 23.3 (22.9)% under a forward (reverse) scan for devices containing the new interconnecting layer, compared to a champion PCE of 18.9 (19.7)% for the reference devices, along with enhanced operational stability, retaining the initial PCE after 100 h when operated at the maximum power point of the device. This work demonstrates the potential for low- cost, easily deposited materials like GO in an interconnecting layer to enhance device performance and stability of TSCs while also opening up a wider range of HTLs that can be utilized toward the next generation of high-performance photovoltaic devices.

We fabricate tandem perovskite solar cells with a reference architecture containing the ALD-SnO_2_/Au interconnecting layer (Figure 1a). The WBG absorber with nominal composition Cs_0.25_FA_0.75_Pb(I_0.73_Br_0.27_)_3_ has a 1,3-propanediammonium iodide (PDAI_2_) layer on its surface to passivate defects and reduce nonradiative recombination at this interface.^20,43^ The LBG absorber with nominal composition Cs_0.25_FA_0.75_Pb_0.5_Sn_0.5_I_3_ passivated with an ethylenediammonium diiodide (EDAI_2_) layer on the surface.^19^ We assess the performance of a 2PACz HTL in the LBG subcell of the reference tandem architecture by directly substituting PEDOT:PSS with 2PACz (Figure S1), with the aim of enhancing the performance of the LBG subcell as is observed when 2PACz is implemented in single-junction lead–tin devices. However, we observe a reduced *J*_SC_ and *V*_OC_ compared to the reference, indicating that there is poor charge extraction and/or increased nonradiative recombination within the bulk absorber (Figure S1). We hypothesize that this may be due to the chemical incompatibility of 2PACz with the underlying Au interlayer. The introduction of the 2PACz HTL yields better performance than an equivalent HTL-free device, which indicates there is some 2PACz deposition that impacts the performance of the device, yet a significantly lower PCE compared to the reference suggests that the 2PACz layer may be nonuniform.

**Figure 1:**
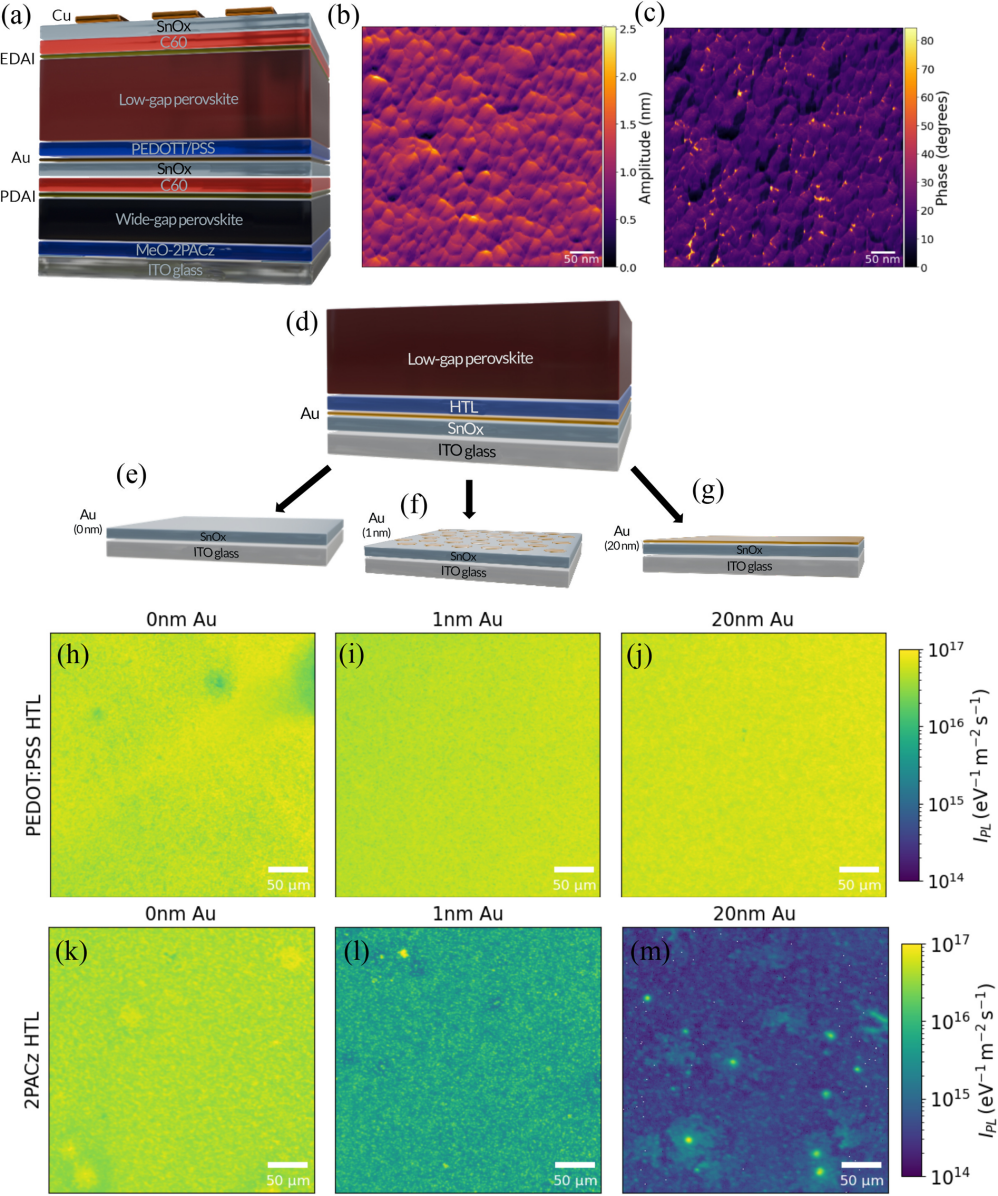
AFM and absolute PL maps of LBG perovskite film deposited on different interlayers. Measurements were carried out at 1-sun intensity with a 405 nm continuous wave laser. (a) Device stack containing the reference interconnecting layer of SnO_2_/Au/PEDOT:PSS. (b) AFM image displaying the amplitude of SnO_2_/1 nm Au interlayer deposited on the WBG subcell. (c) AFM image displaying the phase of SnO_2_/1 nm Au interlayer deposited on the WBG subcell. (d) Stacks used for hyperspectral PL imaging consisting of ITO glass/SnO_2_/0–20 nm Au/HTL/LBG perovskite. (e) Stack containing 0 nm Au. (f) Stack containing 1 nm Au. (g) Stack containing 20 nm Au. (h–j) PL maps of LBG perovskite in stack containing a PEDOT:PSS HTL and 0–20 nm Au. (k–m) PL maps of LBG perovskite in stack containing a 2PACz HTL and 0–20 nm Au.

As the nonuniformity is likely linked to the underlying surface, we carry out atomic force microscopy (AFM) on stacks consisting of WBG subcell/SnO_2_/Au. Figure 1 panels b and c display the AFM amplitude and phase images, respectively. Due to the thin nature of the Au interlayer, it is not possible to visualize the Au on top of the SnO_2_ interlayer in Figure 1b; however, Figure 1c reveals clusters of a different phase than the SnO_2_ surface, which we hypothesize are clusters of Au. We also deposit by thermal evaporation 1 nm of Au directly onto glass and measure AFM. We observe clusters with an amplitude of up to 1 nm and a width on the tens of nanometers scale with a different phase to the glass surface (Figure S2). These measurements confirm that the Au forms a nonconformal layer within the tandem device, leaving areas of SnO_2_ exposed underneath to which the 2PACz can bind. Given the ultrathin nature of the self-assembled monolayer (SAM) formed by 2PACz, its presence cannot be directly measured by using AFM. X-ray photoelectron spectroscopy (XPS) analyses of the C 1s and P 2p signals on samples consisting of ITO glass/SnO_2_/1 nm Au (control) and ITO glass/SnO_2_/1 nm Au/2PACz confirm that SAM molecules are anchored to the surface of the sample in which 2PACz was deposited (Figure S3). Based on the Sn 3d signal, we observe Sn in both stacks, but the intensity is significantly lower for the sample containing 2PACz. The reduced intensity of the Sn peaks is consistent with 2PACz being bound to the SnO_2_ layer and partially blocking the signal detected in the surface region. We also observe a shift to higher binding energies compared to the control, indicating that 2PACz is interacting with the SnO_2_. The analysis of the Au 4f region shows the presence of Au peaks in both samples, with no observable shift in binding energy (Figure S3). These data support the proposition that there is 2PACz bound to the SnO_2_/Au interlayer, though its spatial distribution and continuity is not yet clear.

To investigate the impact of the Au interlayer’s uniformity on device performance, we employed absolute photoluminescence (PL) microscopy. Half-tandem device stacks consisting of ITO glass/SnO_2_/Au/HTL/LBG perovskite were fabricated (Figure 1d). We investigate three Au interlayer thicknesses: 0 nm where the Au interlayer is not incorporated into the stack (Figure 1e), 1 nm as used in the reference interconnecting layer (Figure 1f), and 20 nm in which there is a conformal layer of Au (Figure 1g). We then deposit either PEDOT:PSS or 2PACz as the HTL before spin-coating the LBG lead–tin perovskite. We find that the stacks containing 0 nm of Au in which the HTL, either PEDOT:PSS (Figure 1h) or 2PACz (Figure 1k), is deposited directly onto SnO_2_ have a similar absolute PL value regardless of the HTL implemented. The PL intensity of the stacks containing PEDOT:PSS was found to be independent of the Au interlayer thickness, as there is negligible difference in PL intensity with Au thicknesses between 0 and 20 nm (Figure 1h–j).

However, when a 2PACz HTL was implemented, there is a significant drop in absolute PL intensity when the Au thickness increases from 0 nm (Figure 1k) to 1 nm (Figure 1l). In Figure 1m we observe localized areas of higher PL intensity, which may correspond to exposed SnO_2_ regions where the 2PACz HTL preferentially binds. These areas show PL intensities similar to those in the 0 nm Au map, while regions of lower PL intensity may indicate areas where Au deposition is present, limiting 2PACz binding and resulting in a reduction in PL intensity due to nonradiative recombination and/or PL quenching if the perovskite is in direct contact with the Au. When the Au interlayer thickness is increased to 20 nm there is a further decrease in PL intensity, with few regions of high PL, likely as there are few areas of exposed SnO_2_ (Figure 1m) to which the 2PACz can bind. These results highlight why a phosphonic acid–based SAM such as 2PACz is unsuitable to be used within the reference tandem architecture due to its inability to bind to the Au.

We plot the quasi-Fermi level splitting (QFLS) maps of the stacks containing 1 nm Au and find that the spatially averaged QFLS value of the stack containing 2PACz is 0.06 eV lower than that of the stack containing PEDOT:PSS (Figure S4). This value matches the *V*_OC_ reduction of 0.06 V observed between the reference tandem device and the device in which the LBG subcell contains a 2PACz HTL (Figure S1). Therefore, the reduction in absolute PL when a 2PACz HTL is implemented directly correlates to a reduced *V*_OC_ value in the tandem device.

We then investigated another SAM, namely, 9*H*-carbazole-9-hexanethiol (V1440), within the reference device architecture. The presence of a thiol group rather than a phosphonic acid group means that the SAM should be able to bind to the Au.^44^ We repeated the above experiment, this time implementing a V1440 HTL, and fabricated stacks containing either 0, 1, and 20 nm of Au (Figure S5). We observed that the PL intensity displayed the opposite trend to when a 2PACz HTL was implemented, as an increase in absolute PL is observed as the Au coverage increases, likely as the presence of the SAM between the perovskite and Au or SnO_2_ interlayer reduces nonradiative recombination and/or PL quenching. This indicates that V1440 is binding to the Au regions, and the results are consistent with specific requirements for functional groups within the SAM to bind to Au.

A tandem device was fabricated with V1440 as the HTL in the LBG subcell but the resulting PCE was lower than when using 2PACz, achieving only 4.3% (compared to 9.8%) (Table S1). This result could stem from the Au interlayer consisting of small clusters, which may limit the V1440 deposition to these regions alone. To promote a more uniform self-assembled monolayer (SAM), we aimed for selective binding: V1440 to the Au clusters and 2PACz to the exposed SnO_2_ by depositing both SAMs. We still observe a low PCE (<5%). We summarize the results from the *J*–*V* scan in Figure S6 and Table S1. If the Au recombination layer is removed and the 2PACz HTL is deposited directly on the SnO_2_ layer, to which it can bind, an s-shaped current–voltage curve is observed (Figure S7) attributed to the charge accumulation effect due to the low work function of the n-type SnO_2_ and high work function of the p-type 2PACz HTL.^10^ These results reiterate the need to implement a conductive recombination layer to enable fast extraction of carriers and ensure that the device can achieve a high FF.

Alongside the requirements of conductivity, to implement 2PACz as the HTL within the LBG subcell, it is necessary to use a recombination layer, to which the phosphonic acid group can bind. To do so, we explored graphene oxide (GO) as an alternative recombination layer. GO is dispersible in water and is hydrophilic, and thus it is suitable for solution processing.^45^ There is therefore no risk of sputter damage, as would be with ITO, and there is a potentially reduced cost compared with the thermal evaporation of Au. GO has a mixture of sp^2^- and sp^3^-hybridized carbon atoms, covalently bonded to oxygen-containing functional groups (=O, −OH, −O–, −COOH) on the basal plane or the edges. We hypothesized that we could then replace the PEDOT:PSS HTL within the LBG subcell with a 2PACz HTL, due to the abundance of oxygen-containing functional groups of GO to which the 2PACz could bind. We carried out UV–visible (UV–vis) spectroscopy on tandem stacks consisting of the WBG subcell and the interconnecting layer (i.e., ITO glass/WBG subcell/SnO_2_/recombination layer/HTL) to compare the transmission of light through the WBG subcell and three distinct interconnecting layers; SnO_2_/Au/PEDOT:PSS (referred to as the reference), SnO_2_/GO/PEDOT:PSS (referred to as GO/PEDOT:PSS) and SnO_2_/GO/2PACz (referred to as GO/2PACz). We observe that when the Au recombination layer is replaced with GO, there is a significant increase in the transmission in the infrared region. This is increased further when the PEDOT:PSS HTL is replaced with 2PACz (Figure 2a). We therefore look to implement SnO_2_/GO/2PACz into the tandem device with the aim of reducing parasitic light absorption.

**Figure 2:**
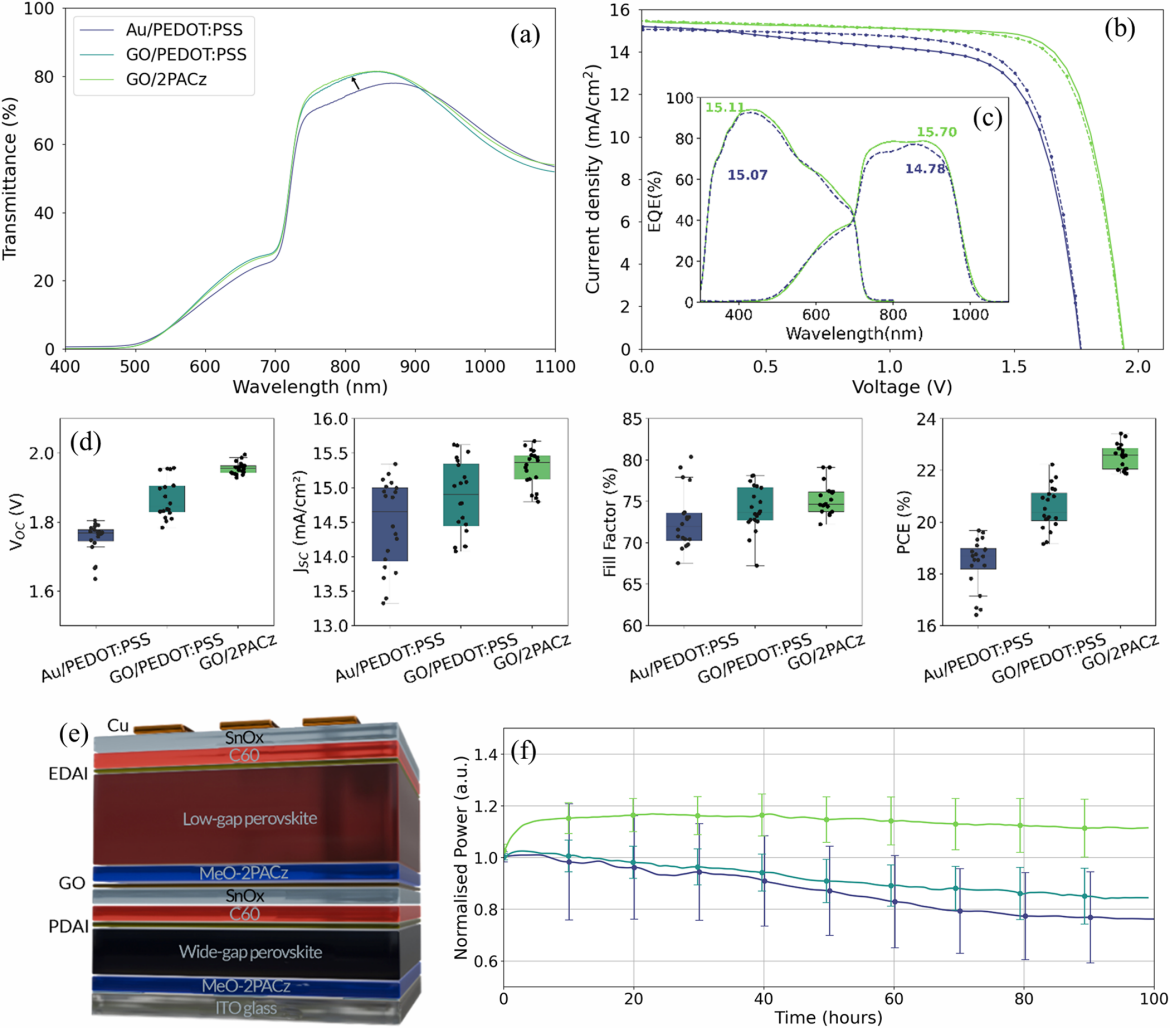
Performance characteristics of tandem devices with three distinct interconnecting layers. (a) Transmittance spectra of device stacks consisting of WBG subcell and one of the interconnecting layers (three architectures are compared: SnO_2_/Au/PEDOT:PSS, SnO_2_/GO/PEDOT:PSS and SnO_2_/GO/2PACz). (b) *J*–*V* curve of champion reference device and champion device with SnO_2_/GO/2PACz interconnecting layer. (c) EQE spectra of reference tandem device and device with SnO_2_/GO/2PACz interconnecting layer, with the integrated current (mA/cm^2^) noted on each curve. (d) Statistical summary of the *J*–*V* performance metrics (*V*_OC_, *J*_SC_, FF and PCE) of 10 devices for each of the three architectures. (e) Tandem stack of the device containing the GO/2PACz interconnecting layer. (f) Maximum power point tracking of 10 devices of each of the three architectures. Measurements carried out under N_2_ at 25 °C. We plot the mean normalized PCE of the devices alongside the standard deviation for the 10 devices.

We fabricate and optimize GO/2PACz tandem devices. In order to achieve the highest PCE, the optimum concentration of the GO recombination layer was found to be 0.35 mg/mL, largely due to this concentration leading to the highest FF (Figure S8). Based on AFM measurements, we approximate this thickness to be below 5 nm but cannot obtain a more precise value. Below this concentration, the GO layer appears to be too thin, and we see a reduction in *V*_OC_, *J*_SC_, and FF, which we attribute to a nonuniform GO layer. Above this concentration, we see a reduction in FF, likely due to the lower conductivity of a thicker GO layer. We then fabricate three types of tandem devices with the interconnecting layer architectures listed above. Statistical analyses of current–voltage (*J*–*V*) characteristics of both the forward and reverse scans of 10 devices of each architecture are displayed in Figure 2d. Overall, the PCE of the GO-containing devices is significantly increased, with the median PCE increasing from 18.7% for the reference device to 20.4% when the Au recombination layer is replaced with GO. The median PCE is increased further to 22.6% when the PEDOT:PSS HTL is replaced with 2PACz. The champion device of each interconnecting layer architecture is displayed in Table 1, with the champion PCE of the reference, GO/PEDOT:PSS and GO/2PACz devices achieving 18.9 (19.7)%, 20.9 (22.2)% and 23.3 (22.9)%, under a forward (reverse) scan, respectively (Figure 2b). The improvement in PCE between the reference and GO/2PACz devices is attributed to increases in all *J–V* parameters *J*_SC_, *V*_OC_, and FF. The median *J*_SC_ is improved from 14.6 to 15.4 mA/cm^2^ compared to the reference device, which is attributed to the reduced parasitic absorption. There is also an increase in median *V*_OC_ from 1.77 to 1.96 V and an improvement in FF from 71.9 to 74.6%. We compare the median PCE of devices with different architectures produced in the same batch across 9 batches (Figure S9). In 8 out of 9 batches, devices incorporating the GO recombination layer achieve a higher median PCE. Specifically, devices with the GO/2PACz interconnecting layer outperform the reference in 6 batches, while those with the GO/PEDOT:PSS layer achieve a higher PCE in 7 batches. This comparison provides a realistic evaluation of device architectures as variations in PCE across batches are expected when fabricating laboratory-scale all-perovskite tandem devices. Overall, the GO interlayer demonstrates improved reproducibility when implemented alongside either a 2PACz or a PEDOT:PSS HTL.

**Table 1:** Champion PV Performance Metrics of the Tandem Devices with Three Distinct Interconnecting Layers from the Forward (Reverse) *J*–*V* Scan

Interconnecting layer	*V*_OC_ (V)	*J*_SC_ (mA/cm^2^)	FF (%)	PCE (%)
Au/PEDOT:PSS	1.77 (1.77)	15.2 (15.1)	70.4 (73.7)	18.9 (19.7)
GO/PEDOT:PSS	1.87 (1.85)	15.4 (15.5)	76.8 (72.5)	22.2 (20.9)
GO/2PACz	1.94 (1.94)	15.4 (15.5)	77.7 (76.2)	23.3 (22.9)

Due to the TSCs with the GO/2PACz interconnecting layer achieving the champion PCE, we focus our analysis on this architecture. The tandem stack is displayed in Figure 2e. We measure the external quantum efficiency (EQE) of the tandem devices and observe that the increased light transmission to the LBG subcell with the new GO/2PACz interlayers compared to the reference (Figure 2a) results in a higher *J*_SC_ in the LBG subcell (Figure 2c), and therefore a consistently higher *J*_SC_. Alongside the increase in PCE, we also observe an improvement in stability for the devices containing the GO interlayer. We carry out maximum power point (MPP) tracking in N_2_ of 10 devices of each architecture and find that on average after 100 h the tandems with the GO/2PACz interconnecting layer still retains at least their initial PCE after an initial efficiency rise. We also carried out MPP tracking of over 25 devices of each architecture for 20 h and observed the same trend over this time region (Figure S10).

We also assessed the *V*_OC_ losses between the reference and GO/2PACz TSCs. We selectively probe each subcell of the champion devices using hyperspectral absolute PL imaging at 1-sun excitation intensity. Using the high-energy slope of the PL spectra, we obtain the quasi-Fermi level splitting (QFLS) maps of the subcells.^46^ Measuring the QFLS of perovskite films and solar cells has been proven to be an efficient approach to assess the recombination losses, as the QFLS can estimate the internal voltage (Δμ).^47−51^ We compare the subcell QFLS values to *V*_OC,rad_ for each subcell, based on the Shockley–Queisser limit for each bandgap, and compare the nonradiative QFLS losses.^52^ We estimate the bandgap (*E*_g_) of the tandem subcells from the EQE spectra by locating the maximum in the spectra derivative and determining the inflection point of the EQE spectrum (Figure S11).^53^ Using this methodology, we estimate the *E*_g_ of the WBG subcell to be 1.74–1.75 eV, while the *E*_g_ of the LBG subcell is between 1.27 and 1.28 eV, which we confirm by using the sigmoid parametrization (Figure S12). The similar bandgap estimated for each device architecture indicates that the different interconnecting layers do not affect the subcell bandgaps. We obtained the QFLS maps for each subcell in both the reference and the GO/2PACz tandem (Figure 3a,b). The spatially averaged QFLS values were obtained for each subcell (Figure S13), and we find, using these values, that the QFLS of the LBG subcell in the GO/2PACz tandem lies 0.11 eV below *V*_OC,rad_, while the reference tandem is 0.14 eV below *V*_OC,rad_ (Figure 3c). The QFLS of the WBG subcell of the GO/2PACz tandem lies 0.14 eV below *V*_OC,rad_, while the reference tandem is 0.19 eV below (Figure 3d). Therefore, for both types of tandem architecture, the greatest loss of QFLS results from the WBG subcell. The QFLS values of both subcells within the reference device are lower than that of the GO/2PACz device, which may be due to greater amounts of nonradiative radiation. We plot histograms of the distribution of the QFLS of each subcell and find that the distributions in the LBG subcell (Figure S14) and WBG subcell (Figure S15) are more homogeneous in the GO/2PACz tandem than in the reference.

**Figure 3:**
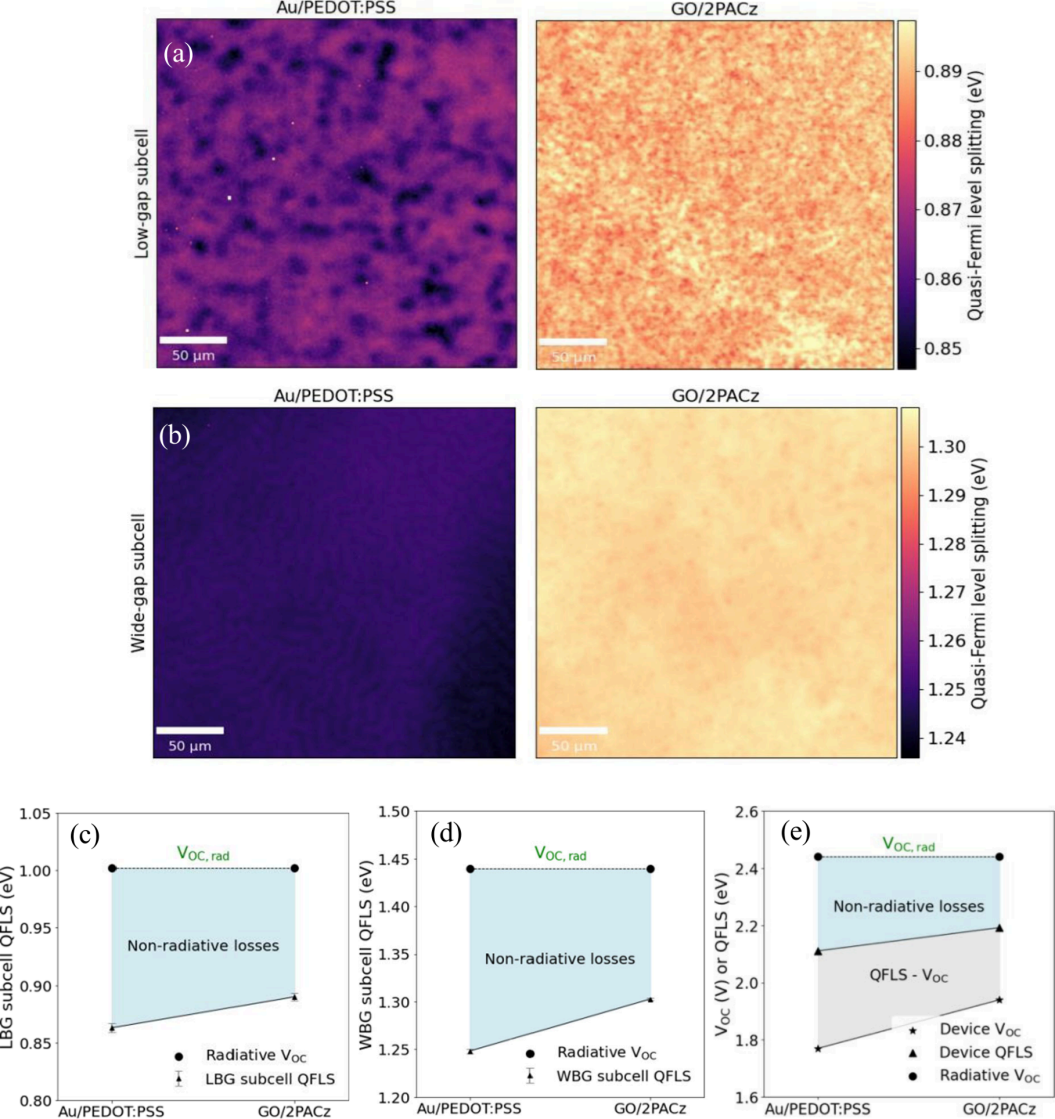
Quantifying QFLS losses in tandem devices with the reference and GO/2PACz architecture. (a) QFLS maps of the LBG subcell of the reference and the GO/2PACz device. (b) QFLS maps of the WBG subcell of reference and GO/2PACz device. (c) Comparison of the spatially averaged QFLS values of each subcell compared to *V*_OC,rad_ for the LBG subcells and (d) the WBG subcells. Error bars represent the standard deviation of the QFLS values obtained. (e) Comparison of total device QFLS, calculated from the sum of the individual subcell QFLS, compared to the *V*_OC_ and *V*_OC,rad_ for both devices.

We then compare the total device QFLS (calculated from the sum of the subcell QFLS values) to the device *V*_OC_. Theoretically, the QFLS and *V*_OC_ are two interchangeable quantities that are equal to each other.^54^ However, as the *V*_OC_ is measured at the external contacts and the QFLS is an internal quantity of the absorber layer, it is commonly experimentally observed that the internal QFLS and external *V*_OC_ are not equal.^47,55^ We find that, for both devices, the total device QFLS is significantly higher than that of the device *V*_OC_. This is generally attributed to misalignment in energy levels between the perovskite and the charge transport layers.^55^ In literature, this has been found to be reduced when replacing PEDOT:PSS with 2PACz, providing a more energetically aligned interface.^41^ There may also be additional losses at the device’s external contacts. We then calculate the difference between the total device QFLS (internal *V*_OC_) and the device *V*_OC_ (external *V*_OC_), found to be 0.34 eV for the reference tandem and reduced to 0.25 eV for the GO/2PACz tandem (Figure 2e). Therefore, the QFLS losses resulting from nonradiative recombination, as well as the losses between the internal and external voltage, are reduced when a GO/2PACz interconnecting layer is implemented.

We also carry out morphological characterization of the perovskite deposited on the three distinct interconnecting layers. We fabricate perovskite stacks consisting of the WBG subcell/interconnecting layer/LBG perovskite and carry out scanning electron microscopy (SEM) of the LBG perovskite layer (Figure S16). Histograms of the grain area distribution (Figure S17) reveal that the LBG perovskite deposited on the GO/2PACz interconnecting layer has a greater proportion of larger grains and a mean area of 0.24 μm^2^, compared to the LBG perovskite deposited on the reference and the GO/PEDOT:PSS interconnecting layers which both have a reduced frequency of larger grains and a smaller mean grain area of 0.21 μm^2^. The increased grain size for the perovskite deposited on the GO/2PACz interconnecting layer and not the GO/PEDOT:PSS interconnecting layer implies that this is due to the presence of the 2PACz HTL and is not influenced by the GO interlayer. The reduced number of grain boundaries may be a factor in improving the PCE and stability of devices with GO/2PACz devices. Grain boundaries can be sources of high defect densities which may induce greater amounts of nonradiative decay than grain interiors, while also providing charge accumulation sites that can trigger the degradation of perovskite.^56^ We carried out X-ray diffraction (XRD) (Figure S18) on the stacks and find that the XRD signals corresponding to the LBG subcell 100, 111, and 200 planes exhibit increased intensity when a GO interconnecting layer is implemented. The intensity of the 100 plane increases further when the PEDOT:PSS HTL is replaced with 2PACz, indicating that the improved perovskite crystallinity is attributed to both the GO and 2PACz layers.

We then studied the ideality factor (*n*_*i*d_) by performing intensity dependent *V*_OC_ (Suns-*V*_OC_) and intensity dependent QFLS (Suns-QFLS) measurements of the tandem devices. As the *V*_OC_ is measured at the external contacts, we refer to the ideality factor obtained from Suns-*V*_OC_ measurements as *n_i_*_d,ext_. It is often reported that *n*_*i*d_ = 1 refers to ideal band-to-band recombination and *n*_*i*d_ = 2 for Shockley–Read–Hall (SRH) recombination. However, when there is trap-assisted recombination with one pinned charge carrier density, for example, traps at the perovskite-transport layer interface, then *n*_*i*d_ = 1.^57^ Therefore, it is important to interpret the ideality factor while taking other parameters into account, as the dominant recombination process cannot be concluded from the ideality factor alone. We estimate that the tandem *n*_*i*d,ext_ of the reference device is 2.78, while that of the GO/2PACz device is 2.66. Figure 4a displays the plot of the Suns-*V*_OC_ measurements while the summary of the estimated ideality factors is displayed in Table 2. In a tandem device, the ideality factor obtained from the Suns-*V*_OC_ slope should approximately equal the sum of those of each subcell,^51^ and so the average ideality factor for the subcells in both tandem devices is <1.5 and we can conclude that recombination is dominated by first-order processes such as band-to-band or interfacial recombination. As shown in Figure 2, the reference device exhibits lower *V*_OC_ and QFLS values, indicating higher nonradiative recombination, which suggests that the first-order processes dominating the reference device involve a higher degree of interfacial recombination compared to the GO/2PACz device, which has a higher degree of radiative recombination.

**Figure 4:**
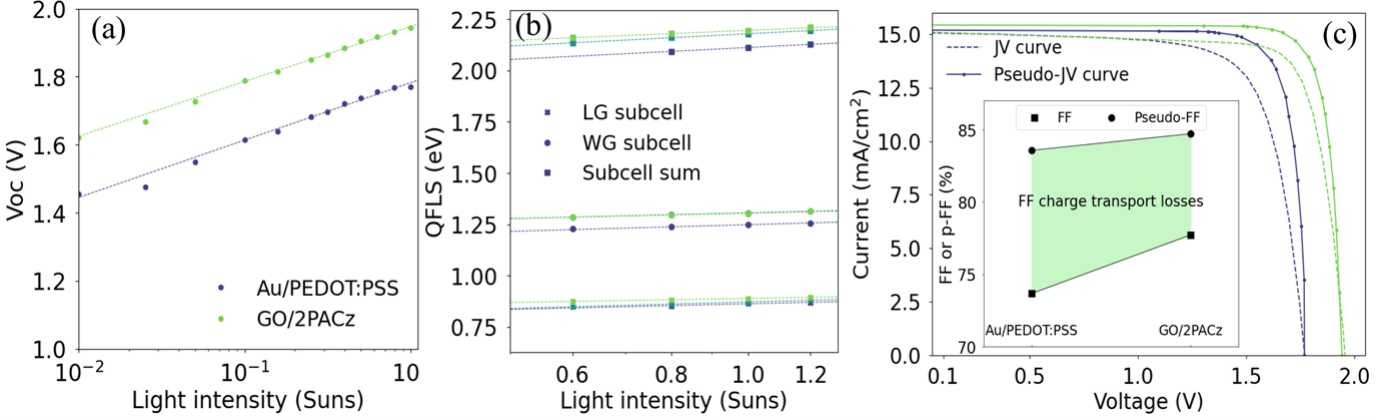
Intensity dependent measurements of reference and GO/2PACz devices. (a) *V*_OC_ as a function of light intensity between 0.01 and 1 Sun. (b) QFLS as a function of light intensity between 0.6 and 1.2 Sun of the LBG and WBG subcell of each device, as well as the total QFLS obtained from the sum of both subcells. (c) Pseudo-*J*–*V* and electrical *J*–*V* plot of both devices. (d) FF charge transport losses obtained by the difference between the pseudo-FF and electrical FF.

**Table 2:** Intensity Dependent *V*_OC_ and QFLS Measurements of Reference and GO/2PACz Device

Interconnecting layer	LG subcell *n*_*i*d,int_(Suns-QFLS)	WG subcell *n*_*i*d,int_(Suns-QFLS)	Tandem *n*_*i*d,int_(Suns-QFLS)	Tandem *n*_*i*d,ext_(Suns-V_oc_)	pFF (%)
Au/PEDOT:PSS	1.51	1.58	3.10	2.78	83.59
GO/2PACz	1.12	1.46	2.56	2.66	84.72

Suns-*V*_OC_ measurements alone cannot determine the contribution of each subcell to the ideality factor. To estimate these contributions, we performed Suns-QFLS measurements, displayed in Figure 4b, on each subcell over a range of 0.6 to 1.2 suns (with similar logarithmic relationship holding even at lower sun equivalents, Figure S19). These measurements allow us to extract the internal ideality factor (*n_i_*_d,int_). QFLS is an internal quantity, representing the density of the photogenerated charges recombining at the absorber or its interfaces, not taking into account the recombination processes happening in the transport layers or contacts. Therefore, *n_i_*_d,int_ is primarily influenced by bulk properties and is less impacted by interfacial losses.^47^ A summary of the internal and external ideality factors obtained from Suns-*V*_OC_ and Suns-QFLS measurements is displayed in Table 2. We sum the *n_i_*_d,int_ values for each subcell to obtain the total tandem *n_i_*_d,int_ for each device. We find that the tandem *n_i_*_d,int_ for the reference device is 3.10, which is higher than the *n*_*i*d,ext_ of 2.78. This could indicate that there is significant SRH recombination in the absorber layer and/or could indicate that there are recombination losses occurring at the perovskite surfaces, recombination layer, and/or near the electrodes that affects the *n_i_*_d,ext_ but not *n_i_*_d,int_, resulting in a lower *n*_*i*d,ext_. The sum of the *n_i_*_d,int_ for the subcells of the GO/2PACz device is 2.56. The value of *n_i_*_d,int_ is close to the value of the *n*_*i*d,ext_ of 2.66, which indicates that they are likely dominated by the same recombination mechanism. We then plot pseudo-*JV* (pJV) curves of the devices and obtain the pseudo-FF (pFF), which is the fill factor unaffected by charge transport losses and only impacted by nonradiative losses (Figure 4c). We find that the GO/2PACz device has a slightly higher pFF of 84.7% than the reference device (pFF of 83.6%), indicating reduced FF losses from nonradiative recombination. When the pFF is compared to the real FF obtained from the *J*–*V* curve, there is a greater loss in FF due to charge transport losses for the reference device (9.9%) when compared to the GO/2PACz tandem (7.0%) (Figure 3d).

Overall, from the QFLS measurements of the subcells at 1 sun, and the Suns-*V*_OC_ and QFLS measurements of the tandem devices, we determine that there is reduced nonradiative recombination in the GO/2PACz tandem device compared to the reference. This may be a combined effect of the increased perovskite grain size, improved crystallinity in the LBG absorber, and better interface with the 2PACz than the PEDOT:PSS, as well as a more homogeneous QFLS distribution in the subcells. Alongside the improved transparency of the interconnecting layer, we have been able to obtain enhanced device *V*_OC_, FF and *J*_SC_, resulting in an improved PCE and stability.

Our work presents a new tandem architecture that addresses key limitations of traditional interconnecting layers. The introduction of GO to replace the Au recombination layer facilitates the use of a 2PACz HTL as an alternative to PEDOTL:PSS. We find that PSCs containing the new GO/2PACz interconnecting consistently achieve a higher PCE and improved stability compared to the reference Au/PEDOT:PSS. The champion devices achieved a PCE of 23.4 (22.5)% with the GO/2PACz interconnecting layer and 18.9 (19.7)% with the reference, under a forward (reverse) scan. The substantial increase in PCE is due to a combination of an improved *J*_SC_, *V*_OC_, and FF. We confirmed by UV–visible spectroscopy that the new interconnecting layer enhanced transmission in the infrared region, resulting in an improved *J*_SC_. Moreover, the improvement in *V*_OC_ was investigated using absolute photoluminescence imaging, which revealed an increase in quasi-Fermi level splitting for both subcells when the GO/2PACz interconnecting layer was implemented. This suggests a reduction in nonradiative recombination losses. In particular, the larger grain size and improved crystalline structure observed in the LBG subcell, as confirmed by SEM and XRD analysis, as well as improved interfaces with the HTL (being 2PACz rather than PEDOT:PSS) likely play key roles in these performance improvements. GO is a low-cost, stable material that is easily integrated into a tandem architecture, which is easily deposited by spin-coating in contrast to thermal evaporation of Au. This work not only provides a promising alternative to the Au recombination layer, which will reap cost benefits in future scale up efforts, but also paves the way for the future device optimization and the integration of different HTLs within tandem structures, addressing current challenges associated with conventional interconnecting layers.

## Methods

### All-Perovskite Tandem Fabrication

The perovskite solar cell was deposited on ITO-covered glass substrate (KINTEC Company), which was cleaned using 15 min sonication in a 2% Hellmanex III (Sigma-Aldrich) solution, 5 min in deionized water, 15 min in acetone, and 15 min isopropanol. The substrates were then subjected to 15 min UV/ozone treatment before being transferred to a nitrogen-filled glovebox.

A 10 mM solution of 2PACz (TCI) in anhydrous ethanol was spin-coated (3000 rpm for 30 s, 5 s acceleration), followed by heating at 100 °C for 10 min. The WBG perovskite solution of 0.75 M Cs_0.25_FA_0.75_Pb(I_0.73_Br_0.27_)_3_ was prepared by dissolving cesium iodide (CsI, Sigma- Aldrich), formamidinium iodide (FAI, Greatcell solar), lead iodide (PbI_2_, TCI) and lead bromide (PbBr, TCI) in a 4:1 (vol:vol) mixture of *N*,*N*-dimethylformamide (DMF, Sigma- Aldrich) and dimethyl sulfoxide (DMSO, Sigma-Aldrich). The solution was stirred at 50 °C for 2 h and filtered using a 0.22 μm PTFE membrane before deposition. 100 μL of perovskite was spread on the substrate and deposited by spin-coating (2000 rpm for 10 s with 2 s acceleration and 6000 rpm for 40 s with 4 s acceleration). Anhydrous methyl acetate was dripped onto the spinning substrate 20 s before the end of the program. The substrates were then annealed for 20 min at 100 °C. A 0.5 mg/mL solution of PDAI_2_ (Sigma-Aldrich) was stirred in a 1:1 (vol:vol) mixture of isopropanol and toluene was stirred overnight at 70 °C and filtered using a 0.22 μm PTFE membrane, and spin-coated at 4000 rpm for 20 s, followed by annealing at 100 °C for 5 min.

The substrates were transferred to a thermal evaporator, and 20 nm of C60 (Sigma-Aldrich) was deposited. A 25 nm SnO_2_ interlayer was then deposited by atomic layer deposition (ALD, picosun). Tetrakis(dimethylamino)tin(IV) (TDMASn, EpiValence) was used as a precursor, and H_2_O was used as a reactant. The chamber was heated to 100 °C, and the precursor bubbler was heated to 75 °C. The pulsing sequence consisted of a 0.6 s pulse of TDMASn with a 30 s purge and a 0.1 s pulse of H_2_O with a 30 s purge, resulting in a growth rate of 0.1 nm/cycle. Following ALD for the reference devices, 1 nm of Au was deposited by thermal evaporation. For the devices with the graphene oxide (GO, Graphene Supermarket) interlayer, a 0.5 mg/mL dispersion in water was diluted to 0.35 mg/mL and was spin-coated (3000 rpm for 30 s with 4 s acceleration) and annealed for 10 min at 100 °C.

If a PEDOT:PSS HTL was used, a 3:1 solution (vol:vol) of methanol (Sigma-Aldrich) and PEDOT:PSS (Ossila AI 4083 PEDOT:PSS) was filtered using a nylon membrane and spin-coated on top of the substrates (4000 rpm for 30 s with 3.5 s acceleration). If a 2PACz (TCI) or V1440 (Kaunus University of Technology) HTL was used, then a 1 mM solution was deposited in the same way as the WBG subcell. The LBG perovskite solution of 2 M Cs_0.25_FA_0.75_Pb_0.5_Sn_0.5_I_3_ was prepared by dissolving FAI, CsI, PbI_2_, tin iodide (SnI_2_, Sigma-Aldrich) and tin fluoride (SnF_2_, Sigma-Aldrich) in a 3:1 (vol:vol) mixture of DMF and DMSO. The solution was stirred at room temperature for 2 h and filtered using a 0.22 μm PTFE membrane before deposition. 120 μL of perovskite was spread on the substrate and deposited by spin-coating (4000 rpm for 40 s with 4 s acceleration). We use gas quenching and start blowing N_2_ 30 s before the end of the program for 15–18 s. The substrates were then annealed for 10 min at 120 °C. The substrates were transferred to a thermal evaporator and 20 nm of C60 was deposited. A 25 nm SnO_2_ interlayer was then deposited by ALD at a substrate temperature of 100 °C. The substrates were transferred back to a thermal evaporator, and 120 nm of Cu was deposited.

### Part-Tandem Device Stack Fabrication

The ITO-covered glass substrate was subjected to the same cleaning process as that stated above. The interlayers in all stacks were fabricated as described above. In the stacks used to carry out the UV–vis measurements, the WBG perovskite had a concentration of 1.2 M, as this measurement was carried out prior to optimization of the subcell thicknesses.

### Solar Cell Characterization

The solar cells were measured under 1 sun AM 1.5 G condition using a Sunbrick G2 V LEDs solar simulator with AAA class for all-perovskite tandem solar cells measurement. Current–voltage characteristics were collected by using an Arkeo multichannel platform (Cicci Research). An aperture mask with 0.12 cm^2^ area was used to define the active area. Devices were scanned at a speed of 100 mV/s. We tracked the maximum power point (MPP) using Arkeo multichannel platform under a continuous flow of N_2_ at 25 °C.

### Suns-*V*_OC_ Measurements

Intensity dependent Suns-*V*_OC_ measurements were carried out using neutral density filters to attenuate the light intensity and measuring the resulting current–voltage curve as described above. The slope of the Suns-*V*_OC_ data was used to estimate the external ideality factor *n*_*i*d,ext_. The slope of *n*_*i*d,ext_ can be estimated using
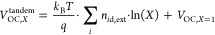
where *n*_*i*d,ext_ denotes the external ideality factor of the individual subcell *i*, *X* denotes “Suns”, *k*_B_ is the Boltzmann constant, *T* is the temperature and *q* is the charge of an electron. The ideality factor of the tandem solar cell *n*^tandem^ is given by the sum of the individual subcell ideality factors:
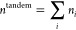
The pseudo-*JV* curve is obtained by
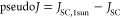
Since the values for *J*_pseudo_ are taken at open-circuit and no current flows in the cell, we can calculate the pseudo FF when there are no charge transport losses.

### EQE

EQE was measured using a Bentham PVE3000 system in transformer mode. A dual xenon–quartz and tungsten halogen lamp were utilized as the light sources, with a swingaway mirror set to 700 nm. A 10 × 10 mm Si reference cell was used to calibrate the power of the probe beam. For WBG (LBG) perovskite, the response scan was obtained from a spectral range of 300–800 (300–1100) nm. An infrared LED bias with 940 nm emission was used for WBG subcell measurement, and a green LED bias with 530 nm emission was used for LBG subcell emission.

### XRD

The XRD patterns were obtained using a Bruker D8 ADVANCE system equipped with a copper-focused X-ray tube (Kα: 1.54 Å) operating at a voltage of 40 kV. During the measurements, the samples were kept in air. The 2θ scan range was from 5 to 55°, with a step size of 0.01° and a dwell time of 0.15 s per step.

### AFM

The atomic force microscopy analysis was conducted on a scanning probe microscope MFP-3D AFM System (Asylum/Oxford Instruments, United Kingdom). Measurements were performed at ambient conditions in tapping mode with an aluminum-coated silicon AFM probe (Tap-150Al-G, BudgetSensors, Bulgaria). Images were obtained on the top surfaces of the dry specimens.

### UV–Vis Spectroscopy

We carry out UV–vis spectroscopy using an Agilent Cary 7000. We used the Diffuse Reflectance Accessory to carry out measurements. We initially carried out a baseline correction using a PTFE reference plate in the reflectance port. We attach the sample to cover the beam and carry out total transmittance measurements across the wavelength range 300–1100 nm. We then moved the sample to the reflectance port and carried out total reflectance measurements.

### SEM

The surface morphology of the perovskite thin films was analyzed with a field-emission scanning electron microscope (ZEISS LEO GEMINI 1530VP FEG-SEM) operating at 2 kV, utilizing an in-lens detector and secondary electron mode. Carbon tape was used to secure the samples onto the holder for observation.

### XPS

XPS analysis was performed by using a Thermo Scientific Escalab 250Xi fitted with a monochromated Al Kα X-ray source (1486.7 eV). All data were recorded with an X-ray beam size of 650 μm and a pass energy of 20 eV at a step size of 0.1 eV. Electronic charge neutralization was achieved using an ion source. Ion gun current = 100 μA. Ion gun voltage = 40 V. All sample data were recorded at a pressure below 10–8 Torr and a room temperature of 294 K. Data were analyzed using CasaXPS v2.3.26rev1.0N.

### Photoluminescence Mapping

Wide-field hyperspectral microscopy measurements were conducted using the Photon etc. IMA system at a magnification of 20×. Luminescence excitation was achieved by using a 405 nm continuous wave laser. The emitted light from the sample was directed onto a volume Bragg grating, which spectrally split the light onto a temperature-controlled CCD camera (Hamamatsu ORCA Flash 4.0 V3 sCMOS camera) operating across a wavelength range of 400–1000 nm. By adjusting the angle of the grating relative to the incident light, spectral data were acquired from each point on the sample. The camera was maintained at 0 °C via a thermoelectric cooler. For the wide bandgap perovskite subcell, images were collected in the 640 to 800 nm range, while the narrow-bandgap perovskite samples were imaged in the 880 to 1000 nm range. A step size of 2 nm was used for all of the measurements. For calibration to determine the absolute number of photons at each point, a two-step process was carried out according to previous literature.^58^ To determine the equivalent number of suns for a monochromatic excitation of a specific power, we used an interpolated AM 1.5G spectrum and converted the spectral irradiance (W m^–2^ nm^–1^) to photons (m^–2^ nm^–1^ s^–1^) by dividing by the photon energy at each wavelength. We then integrated the spectrum over wavelengths from 300 nm to the bandgap energy of the material to obtain the flux of above-bandgap photons. This flux was compared to the photon flux from the monochromatic excitation to calculate the equivalent number of suns.^58^ The power of the laser for 1 sun intensity was 67.9 mW/cm^2^.

### QFLS Determination

To determine the QFLS from the absolute PL spectra, we follow an approach described by Katahara and Hillhouse^59^ and implemented in previous literature.^58^ We write the overall PL intensity, *I*_PL_, as
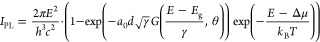
where *E* is energy, *h* is Planck’s constant, *c* is the speed of light, *a*_0_ is a parameter that depends on the oscillator strength of the material, *d* is the thickness of the film, γ is the Urbach energy, *G*(*E* – *E*_g_, θ) is a function in which the convolution integral has been evaluated and γ has been looked up in tables provided by Brady et al.,^60^*k*_B_ is Boltzmann’s constant, *T* is the temperature and Δμ is the QFLS. The *a*_0_*d* product was set as ten for perovskites materials.

We use the Levenberg–Marquardt nonlinear least-squares fitting algorithm implemented in Python to fit the parameters and extract the Δμ values.^58^

### Suns-QFLS

To obtain the Suns-QFLS dependence, absolute PL maps were recorded between 0.6 and 1 suns. All maps spatially averaged the QFLS value was obtained for each intensity. The slope of the Suns- QFLS data was used to find the internal ideality factor *n*_*i*d,int_.
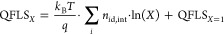
where *n*_*i*d,int_ denotes the internal ideality factor of the individual subcell.

### Band Gap Calculation from the EQE Spectrum

In the EQE spectrum, a smooth absorption threshold occurs with a shape that resembles a sigmoid function. The bandgap can be found from the inflection point of the EQE spectrum by locating the maximum in the spectra derivative ∂EQE/∂*E* (or ∂EQE/∂λ). The absorption threshold can also be paramatrized as^53^
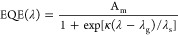
Where κ = ln[7 + 4√3] ≅ 2.63, *A*_m_ and λ_s_ are fitting parameters, *A*_m_ is the maximum EQE just after the step and λ_s_ is the width of the slope just after the step (the distance between the maximum and minimum of the second derivative ∂^2^EQE/∂λ. The position of λ_g_ corresponds to the inflection point of EQE (λ) that is the maximum of the Gaussian-like first derivative ∂EQE/∂λ. When λ_s_ < 100 nm, we can obtain the bandgap value straight from the λ_g_ in the sigmoid parametrization.

## Supporting Information Available

Figures S1–S19 and Table S1 of *J*–*V* data and PL maps of different device architectures containing different interlayer/HTL combinations, AFM and XPS data of Au/2Pacz interlayer, *J*–*V* data summary of optimization of GO thickness, additional stability data of the three different device architectures up to 20 h, determination of bandgap using EQE, analysis of QFLS variation in the LBG subcell, SEM and XRD data of the three different interconnecting layers (PDF)
