# Supplementary material for: Optimized Graphene-Oxide-Based Interconnecting Layer in All-Perovskite Tandem Solar Cells
Source: ACS Energy Lett. 2025 Jan 9;10(2):713–25. doi: 10.1021/acsenergylett.4c03065 (PMC11834147; doi:10.1021/acsenergylett.4c03065)
Supplement: Supplementary file 1 — nz4c03065_si_001.pdf [file nz4c03065_si_001.pdf]

Optimised graphene-oxide-based interconnecting layer in all-perovskite tandem solar cells

Authors

Melissa R. Fitzsimmons,[1] Bart Roose,[1] Yutong Han,[1] Taeheon Kang,[1] Yu-Hsien Chiang,[2] Chieh-Szu Huang,[1] Yang Lu,[1] Terry Chien-Jen Yang,[1,2] Cullen Chosy,[1,2] Shaoliang Guan,[2] Miguel Anaya,[1,2] Samuel D. Stranks*[1,2]

Affiliations

1. Department of Chemical Engineering and Biotechnology, University of Cambridge, Cambridge, CB3 0AS, United Kingdom
2. Department of Physics, Cavendish Laboratory, University of Cambridge, Cambridge, CB3 0HE, United Kingdom

*Email: sds65@cam.ac.uk

Additional Figures

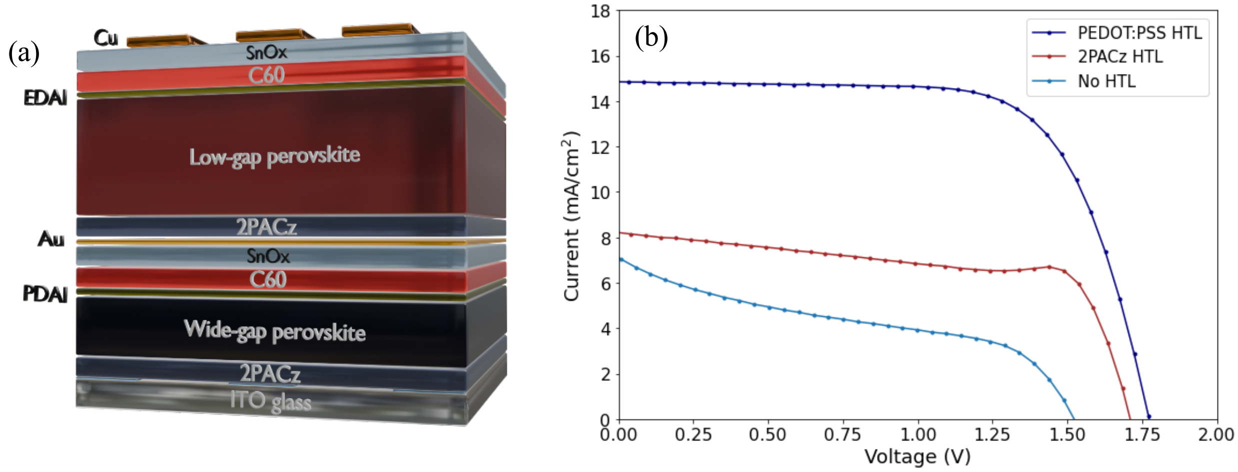

Figure S1. (a) Tandem stack of device containing the reference device architecture with the PEDOT:PSS HTL in the LBG subcell replaced with 2PACz. (b) J-V characteristics of a tandem device using the reference device architecture (navy), 2PACz HTL in the LBG subcell (maroon) and without a HTL in the LBG subcell (blue).

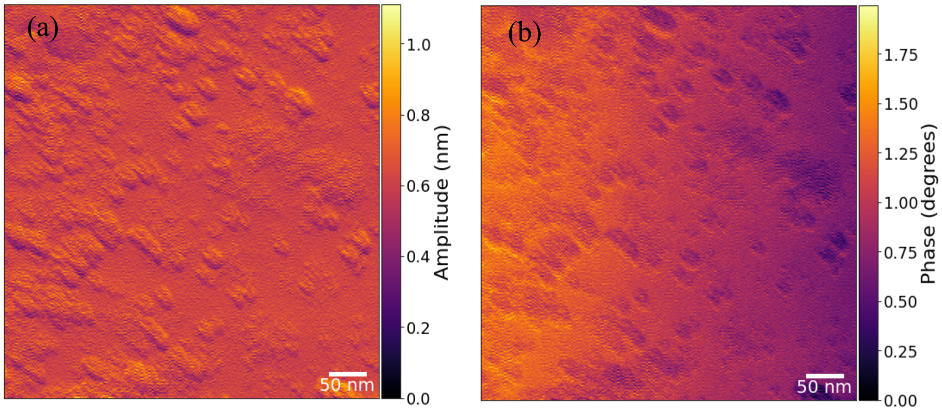

Figure S2. (a) AFM image displaying the amplitude of 1 nm Au deposited on glass. (b) AFM image displaying the phase of 1 nm gold deposited on glass.

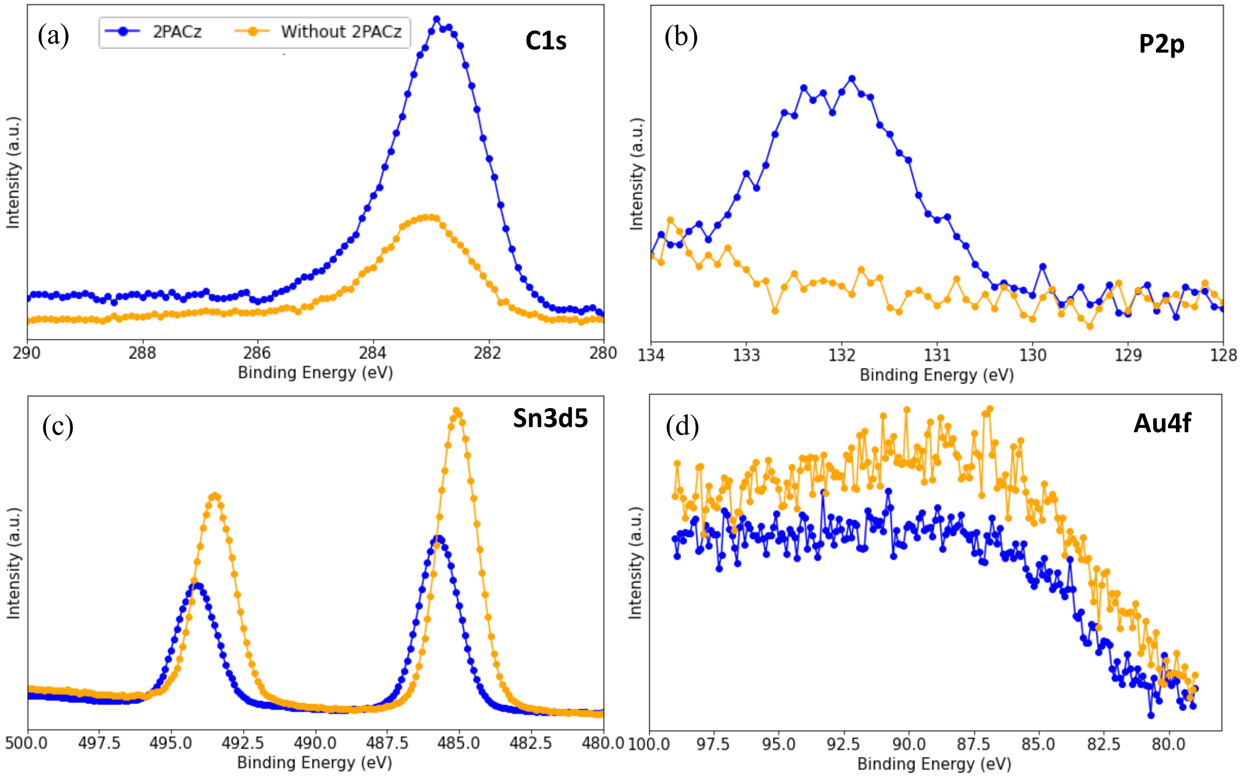

Figure S3. X-ray photoelectron spectra for two stacks: ITO glass/SnO_2/1nm Au/2PACz (blue line) and ITO glass/SnO_2/1nm Au (yellow line) for (a) C1s, (b) P2p, (c) Sn3d5, (d) Au4f.

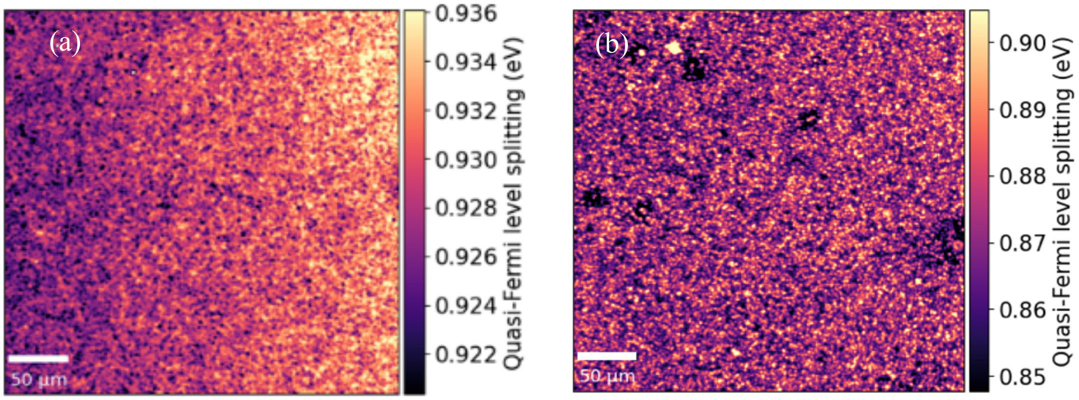

Figure S4. QFLS map obtained from absolute PL map when stack contains PEDOT:PSS HTL deposited on 1 nm Au (Figure 1i), with a spatially averaged QFLS value of 0.93 eV. (b) QFLS map obtained from absolute PL map when stack contains 2PACz HTL deposited on 1 nm Au (Figure 1l), with a spatially averaged QFLS value of 0.87 eV.

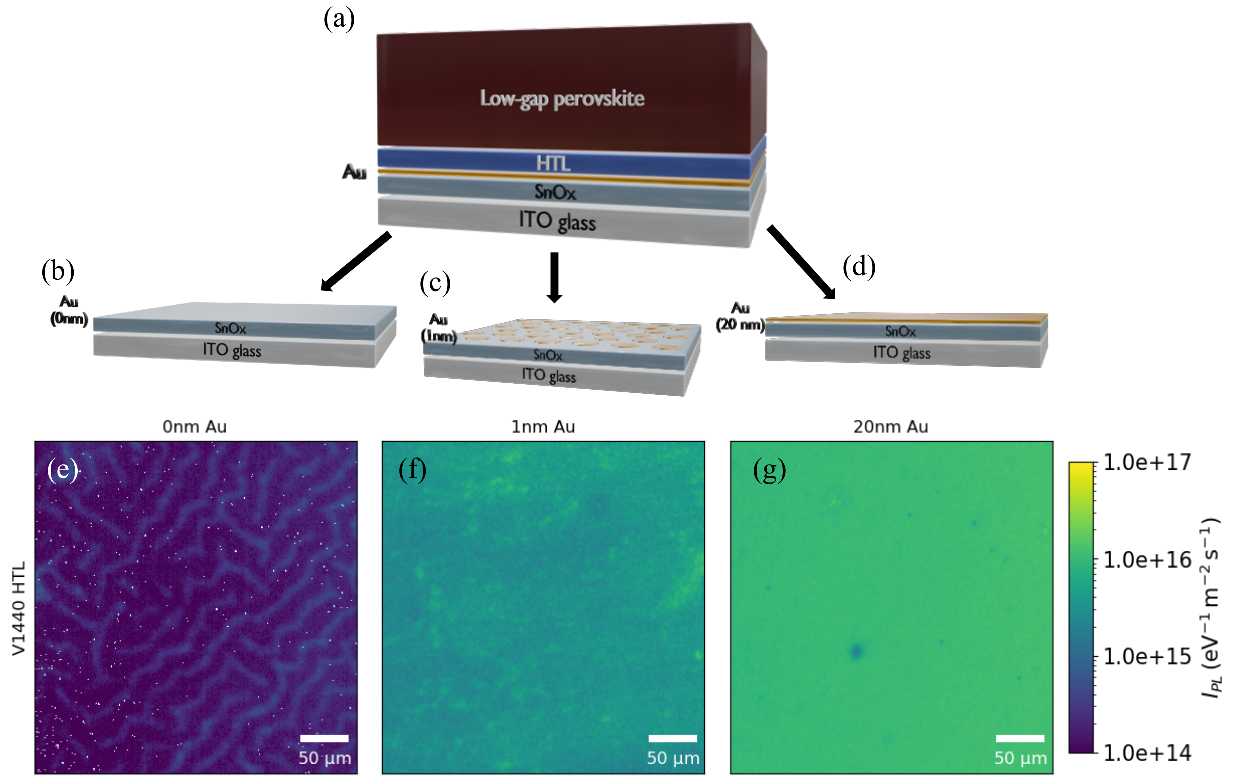

Figure S5. Absolute PL maps of LBG perovskite film. Measurements were carried out at 1 sun intensity with a 405 nm continuous wave laser. (a) Device stack of the stack consisting of ITO glass/SnO_2/0-20 nm Au/V1440/LBG perovskite. (b) Stack containing 0 nm Au. (c) Stack containing 1 nm Au. (d) Stack containing 20 nm Au. (e-g) PL maps of LBG perovskite in stack containing a V1441 HTL and 0-20 nm Au.

Table S1. JV Summary of J-V performance metrics of a tandem device using the reference interconnecting layer containing Au, and different HTLs in the LBG subcell from the reverse J-V scan.

**HTL**	**V_{OC} (V)**	**J_{SC} (mA/cm^2)**	**FF (%)**	**PCE (%)**
PEDOT:PSS (reference)	1.77	15.1	73.7	19.7
2PACz	1.71	8.21	69.4	9.76
V1440	1.58	4.92	55.7	4.33
2PACz and V1440 (same solution)	1.39	5.91	55.2	4.52
2PACz and V1440 (sequential deposition)	1.79	5.49	59.4	5.84
No HTL	1.52	7.22	38.5	4.22

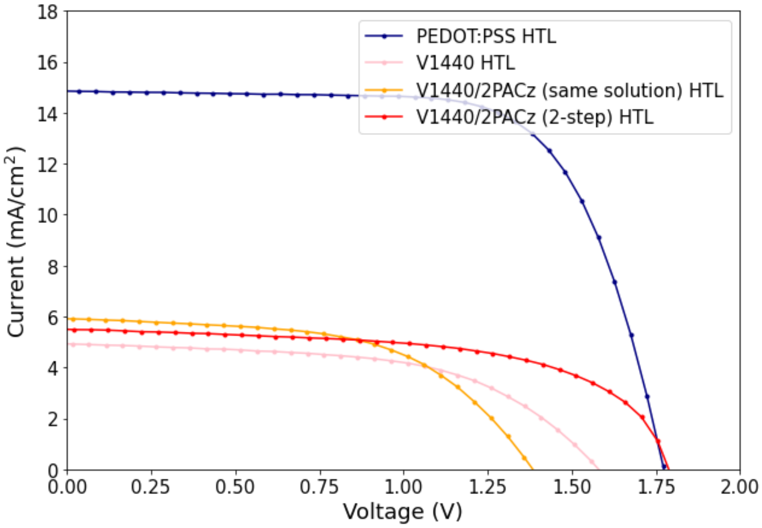

Figure S6. JV Summary of J-V performance metrics of a tandem device using the reference interconnecting layer containing Au, and different HTLs in the LBG subcell from the reverse J-V scan. The HTLS used are PEDOT:PSS (the reference) (navy), V1440 (pink), V1440 and 2PACz after mixing both SAMs in the same solution (yellow) and V1440 and 2PACz deposited sequentially in a 2-step deposition process (red).

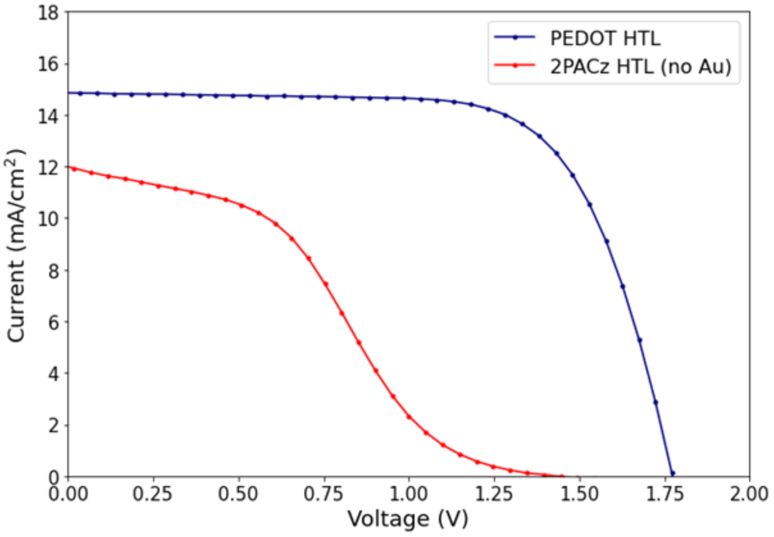

Figure S7. JV Summary of J-V performance metrics of a tandem device using the reference tandem structure (navy) and the device without a recombination layer (red) from the reverse J-V scan.

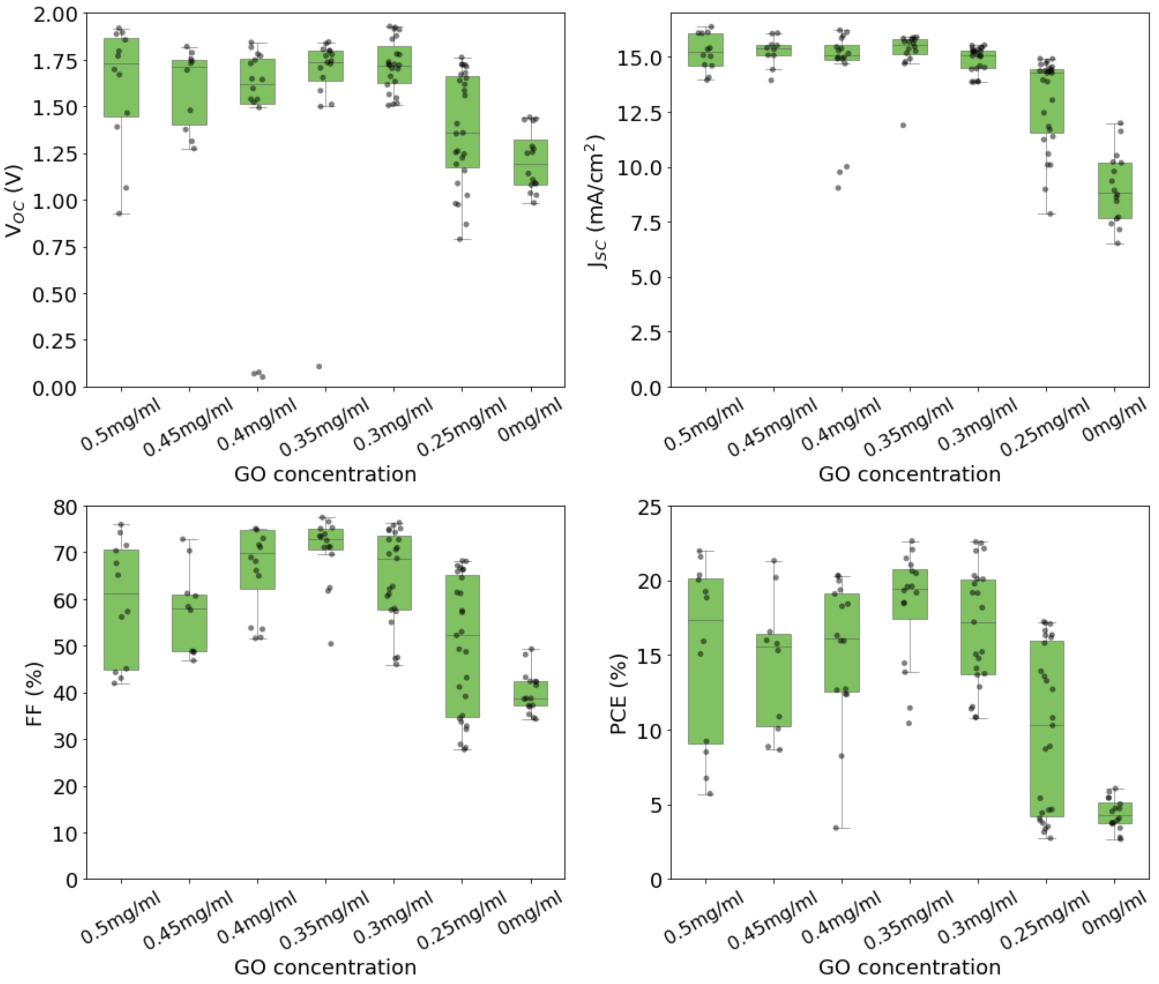

Figure S8. J-V data of the forward and reverse scan of tandem with a GO/2PACz interconnecting layer. GO interlayer concentrations range from 0.0 mg/ml to 0.5 mg/ml GO aqueous solution.

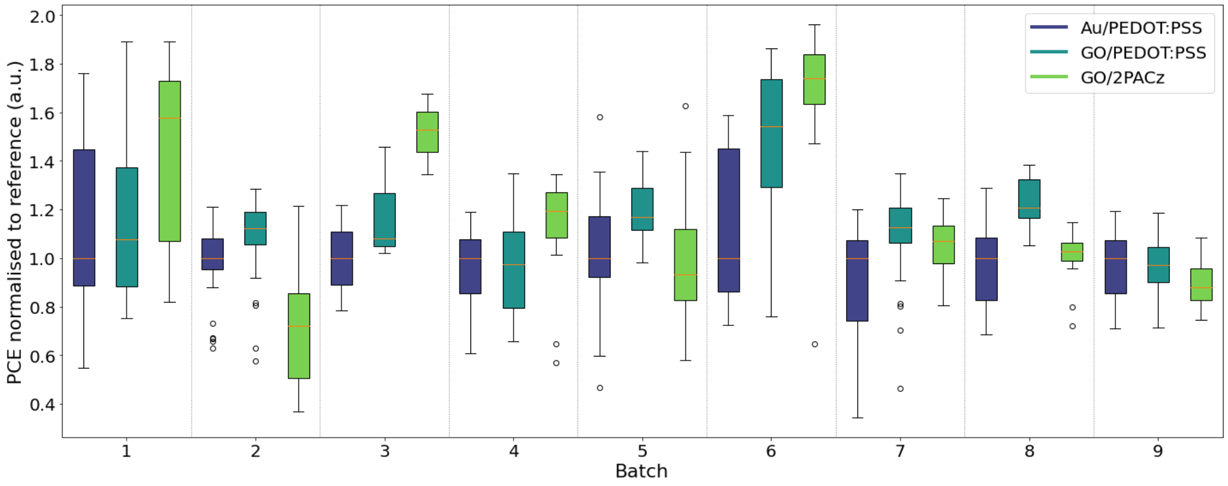

Figure S9. Comparison of median PCE of devices of each architecture produced in the same batch. PCEs are normalised with the PCE of the reference (Au/PEDOT:PSS) normalised to 1. We remove poor batches of devices (in which the median PCE of devices < 10%) as well as remove individual devices with a PCE < 5% from the analysis.

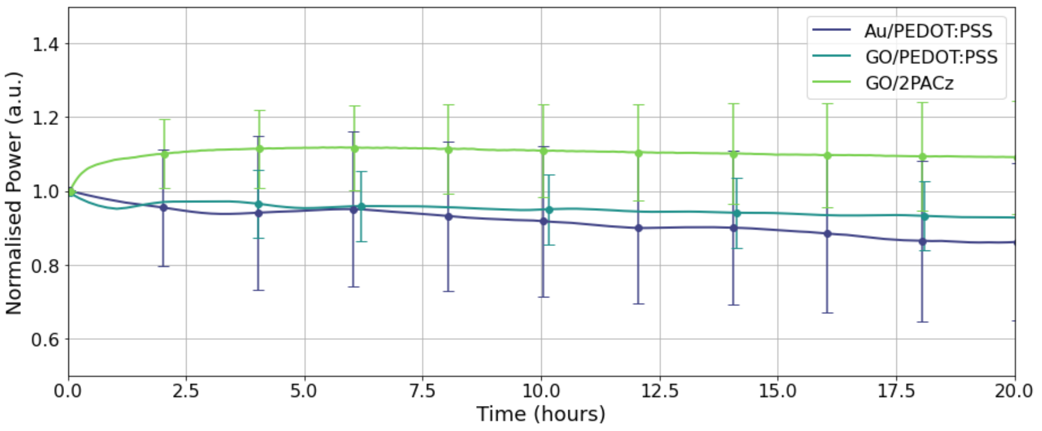

Figure S10. Maximum power point tracking of ≥ 25 devices of each of the three architectures, stability measurements carried out under N_2 at 25°C. We plot the mean normalised PCE of the devices alongside the standard deviation over 20 h.

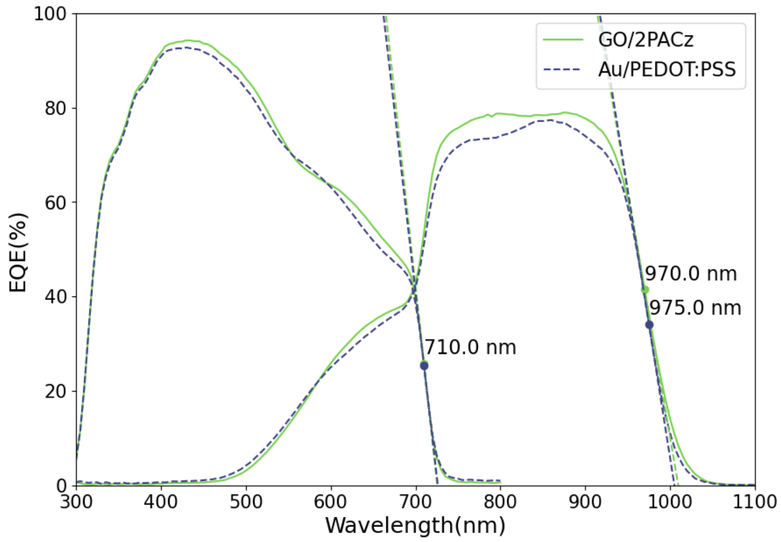

Figure S11. Determination of bandgap from the EQE spectrum. The E_g of the WBG subcells for both interconnecting layer architectures is estimated to be 1.746 eV. The E_g of the LBG subcell for the reference architecture is estimated to be 1.272 eV and for the GO/2PACz interconnecting layer 1.278 eV.

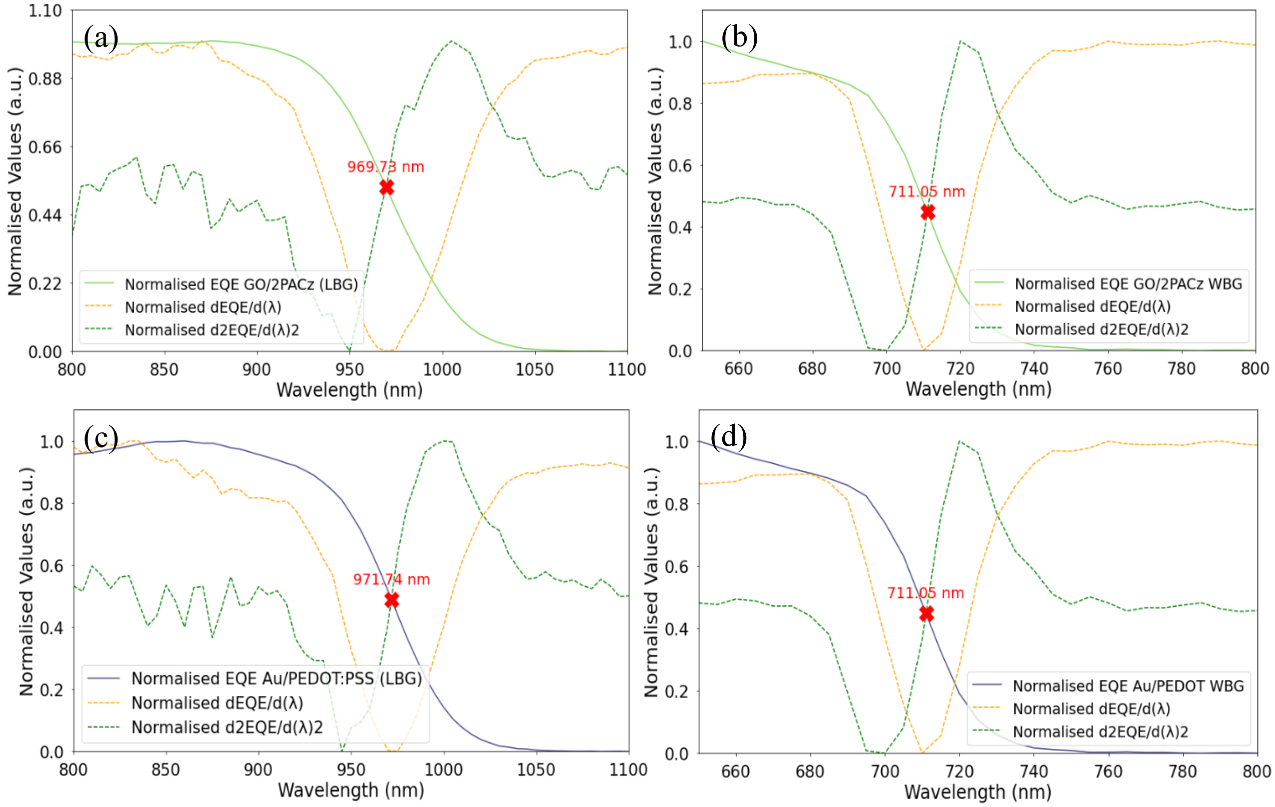

Figure S12. Determination of bandgap through sigmoid parameterisation, displaying the parameters A_m, λ_s and λ_g in a) and mark λ_g (the point in which the second derivative crosses the normalised EQE curve) in all plots. (a) LBG subcell of the GO/2PACz TSC. (b) WBG subcell of GO/PACz TSC. (c) LBG subcell of Au/PEDOT:PSS TSC. (d) WBG subcell of Au/PEDOT:PSS TSC.

We confirm that as $\lambda_s < 100$ nm, we can obtain the bandgap value straight from the λ_g in the sigmoid parameterisation.

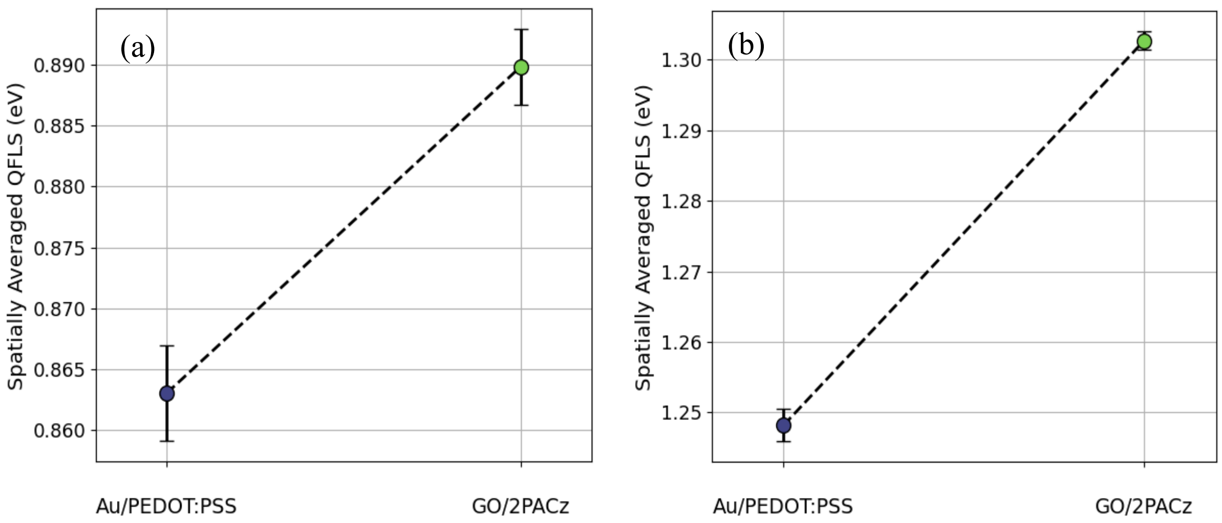

Figure S13. Spatially averaged QFLS values obtained from QFLS maps of each subcell of the tandems with Au/PEDOT:PSS and GO/2PACz interconnecting layer with the standard deviation of the QFLS values plotted as error bars of (a) LBG subcell. (b) WBG subcell.

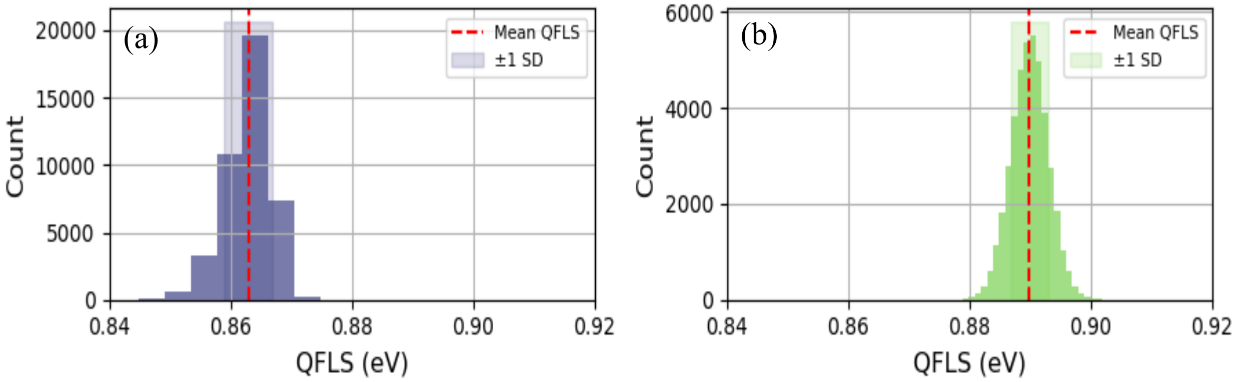

Figure S14. Histogram of QFLS counts in LBG subcell of (a) reference tandem (standard deviation 0.0039 eV) and (b) GO/2PACz tandem (standard deviation 0.0031 eV).

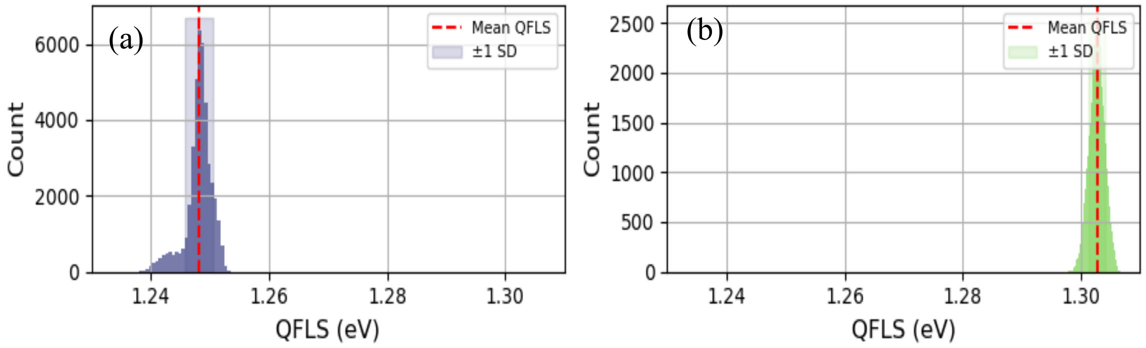

Figure S15. Histogram of QFLS counts in WBG subcell of (a) reference tandem (standard deviation 0.0023 eV) and (b) GO/2PACz tandem (standard deviation 0.0013 eV).

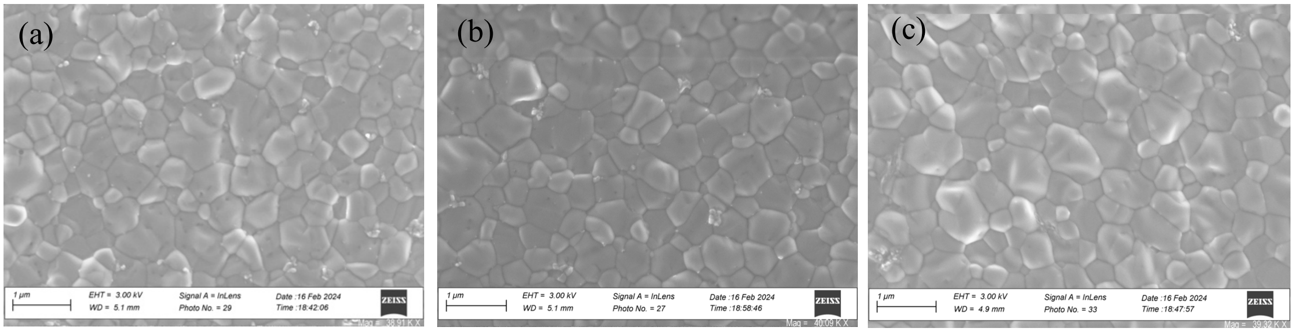

Figure S16. Scanning Electron Microscopy (SEM) images of LBG perovskite deposited on stack consisting of WBG subcell/interconnecting layer/LBG perovskite**.** (a) Reference interconnecting layer. (b) GO/PEDOT:PSS interconnecting layer. (c) GO/2PACz interconnecting layer.

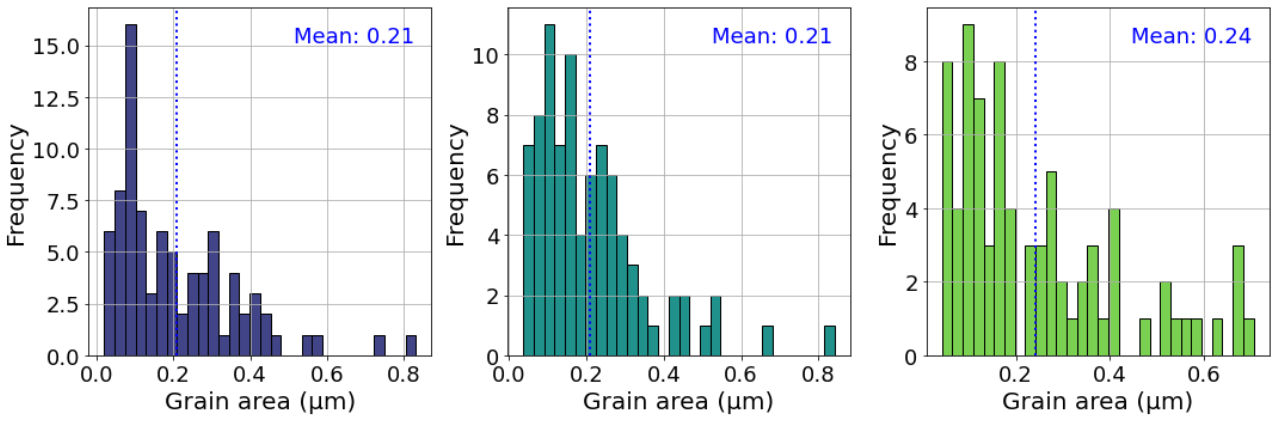

Figure S17. Histogram of grain area distribution calculated from the SEM images in Figure S9a-b. The mean grain size value is displayed on each figure. (a) Reference interconnecting layer. (b) GO/PEDOT:PSS interconnecting layer. (c) GO/2PACz interconnecting layer.

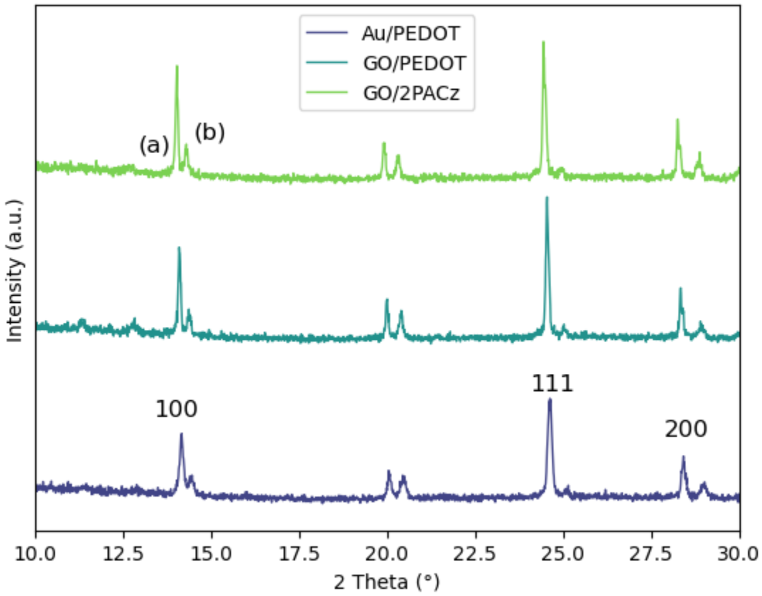

Figure S18. XRD signals of stacks consisting of WBG subcell/interconnecting layer/LBG subcell

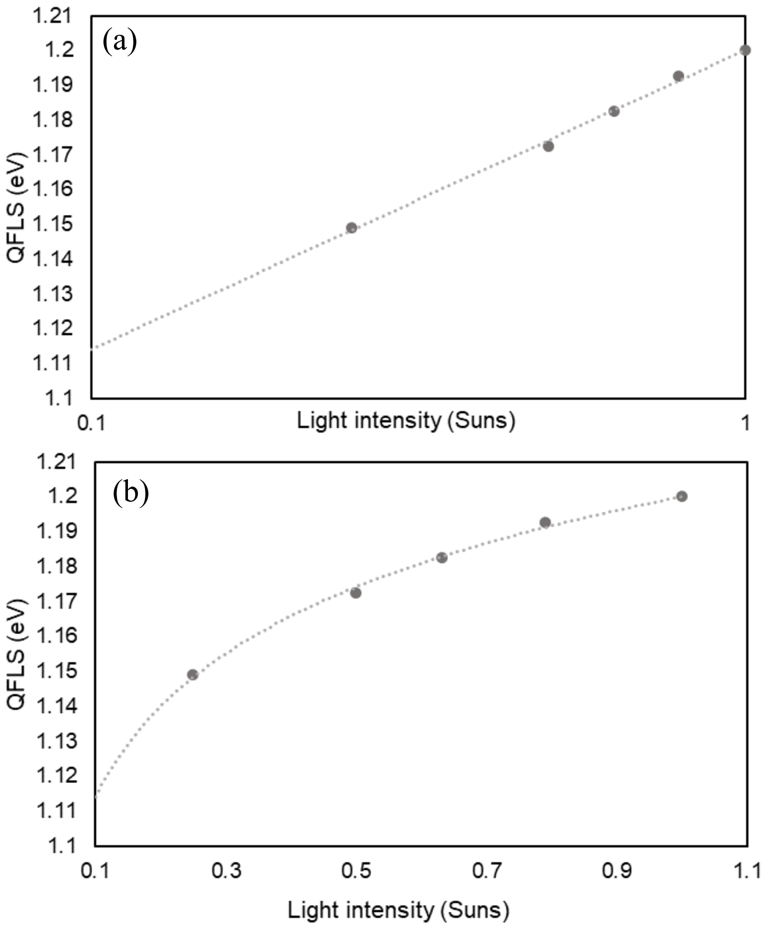

Figure S19. QFLS as a function of light intensity between 0.25 and 1 Sun on the WBG subcell. (a) x-axis on a logarithmic scale. (b) x-axis on a linear scale.
